# Beyond the Flare: A Case of Disseminated Tuberculosis and Thrombosis Masquerading as IBD Exacerbation

**DOI:** 10.1007/s12328-026-02303-6

**Published:** 2026-03-07

**Authors:** Germán Ramírez-Olivencia, Celia Caravaca, Marta Sanz Alba, Álvaro de la Serna Gamboa, Ignacio Díaz Villalonga

**Affiliations:** 1 Faculty of Medicine, Department of Medicine, HLIU and CBRN Infectious Diseases Unit, Gómez Ulla Central Defense Hospital (CSVE), University of Alcalá, Glorieta del Ejército S/N, 28047 Madrid, Spain; 2 Digestive Diseases Department, Hospital Central de La Defensa Gómez Ulla CSVE, Madrid, Spain; 3 Department of Medical Imaging, Hospital Central de La Defensa Gómez Ulla CSVE, Madrid, Spain

**Keywords:** Crohn’s disease, Tuberculosis, Miliary, Venous thrombosis

## Abstract

Differentiating inflammatory bowel disease (IBD) flares from infectious complications in patients on anti-TNFα therapy presents a significant diagnostic challenge. This case report describes a 54-year-old woman with ileocolic Crohn’s disease on adalimumab who presented with systemic and gastrointestinal symptoms mimicking a flare. Initial computed tomography revealed enteritis, mesenteric lymphadenopathy, and extensive inferior vena cava thrombosis. Despite treatment with corticosteroids and broad-spectrum antibiotics, the patient developed respiratory symptoms. Further evaluation confirmed disseminated tuberculosis (TB) through PCR and culture from a bronchoalveolar lavage and histopathological analysis of a lymph node. Adalimumab was discontinued, and the patient was successfully treated with anti-tuberculous therapy and anticoagulation, leading to progressive clinical improvement. This case underscores the importance of a broad differential diagnosis in immunosuppressed IBD patients, as opportunistic infections like TB can mimic IBD flares and require invasive diagnostics for definitive confirmation, especially when systemic symptoms and thrombosis are present.

## Introduction

Crohn’s disease (CD) is a chronic inflammatory bowel disorder characterized by a highly variable clinical course, ranging from intermittent episodes to progressive activity. While primarily presenting with gastrointestinal symptoms such as abdominal pain, diarrhea, and weight loss, CD is frequently associated with extraintestinal manifestations. These include pulmonary involvements—such as bronchiectasis and interstitial or granulomatous lung diseases [1–4] —as well as a significantly elevated risk of venous thromboembolic events (VTE). In fact, the incidence of VTE in patients with inflammatory bowel disease (IBD) is more than double that of the general population, driven by both disease-specific factors, such as inflammatory activity, and external triggers like pneumonia or malignancy [5].

The advent of anti-TNFα agents has revolutionized the management of moderate-to-severe IBD. However, their use introduces diagnostic complexities, most notably an increased susceptibility to active tuberculosis [6–9]. Consequently, distinguishing a true inflammatory flare from opportunistic infections remains a formidable clinical challenge in the context of therapeutic immunosuppression [10, 11]**.**

This report describes a patient receiving adalimumab who presented with disseminated tuberculosis and extensive venous thrombosis, which initially mimicked a CD exacerbation. Our case underscores the necessity of maintaining a broad differential diagnosis and a meticulous diagnostic approach when immunosuppressed IBD patients present with atypical or refractory symptoms.

## Case Report

In 2021, a 54-year-old woman presented with an acute abdomen requiring emergent ileocecal resection, leading to a diagnosis of ileocolic Crohn’s disease (Montreal A3L3B1). Histopathological evaluation confirmed active CD; the specimen demonstrated significant architectural distortion, Paneth cell metaplasia, and chronic transmural inflammation with lymphoid aggregates. Key diagnostic features included multiple non-caseating epithelioid granulomas and crypt microabscesses, with no evidence of malignancy or acid-fast bacilli.

Adalimumab monotherapy was initiated in April 2023 following clinical recurrence. At that time, conventional immunosuppressants (thiopurines) were not an option because of intolerance. Due to subtherapeutic trough levels (6.20 µg/mL in December 2023), the dose was escalated to 80 mg every two weeks in February 2024, achieving adequate serum levels by August 2024 (17.80 µg/mL). Initial screening for latent tuberculosis via Interferon-gamma Release Assay (IGRA) was negative (0.03 and 0.07 UI/ml; cut-off 0.35 IU/mL).

**Fig. 1:**
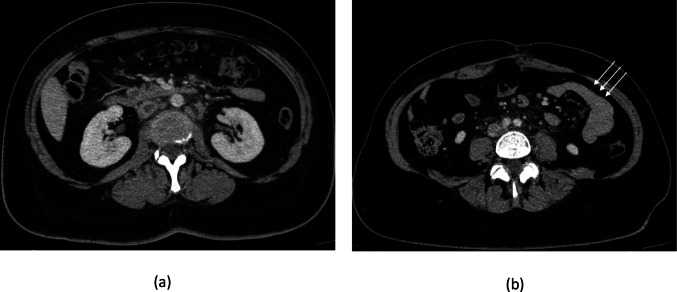
Axial contrast-enhanced abdominopelvic CT shows a central filling defect in the inferior vena cava with retroperitoneal and mesenteric root lymphadenopathy (**a**), and multiple collapsed jejunal loops (arrows) exhibiting mural thickening and peri-enteric fat stranding consistent with inflammatory jejunitis (**b**)

In 2025, the patient presented with a one-month history of fever, diarrhea, and asthenia. An abdominal CT scan revealed jejunal enteritis, mesenteric lymphadenopathy, and extensive thrombosis of the inferior vena cava (Fig. 1). While these findings initially suggested an IBD flare, prompting the acute administration of systemic corticosteroids, antibiotics, and therapeutic anticoagulation, the clinical course dictated a reassessment.

**Fig. 2:**
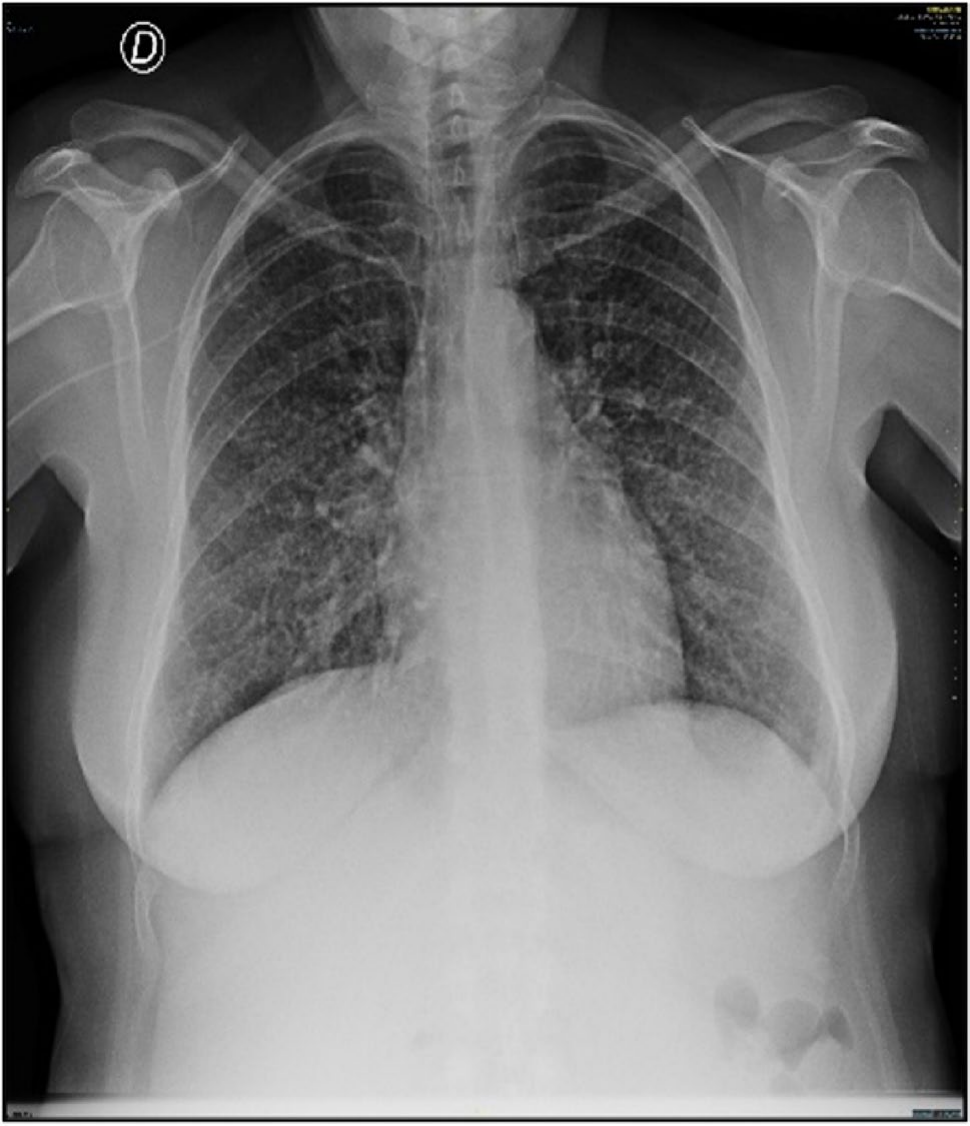
Bilateral ground-glass opacities, predominantly at the lung bases, were evident on the chest X-ray

**Fig. 3:**
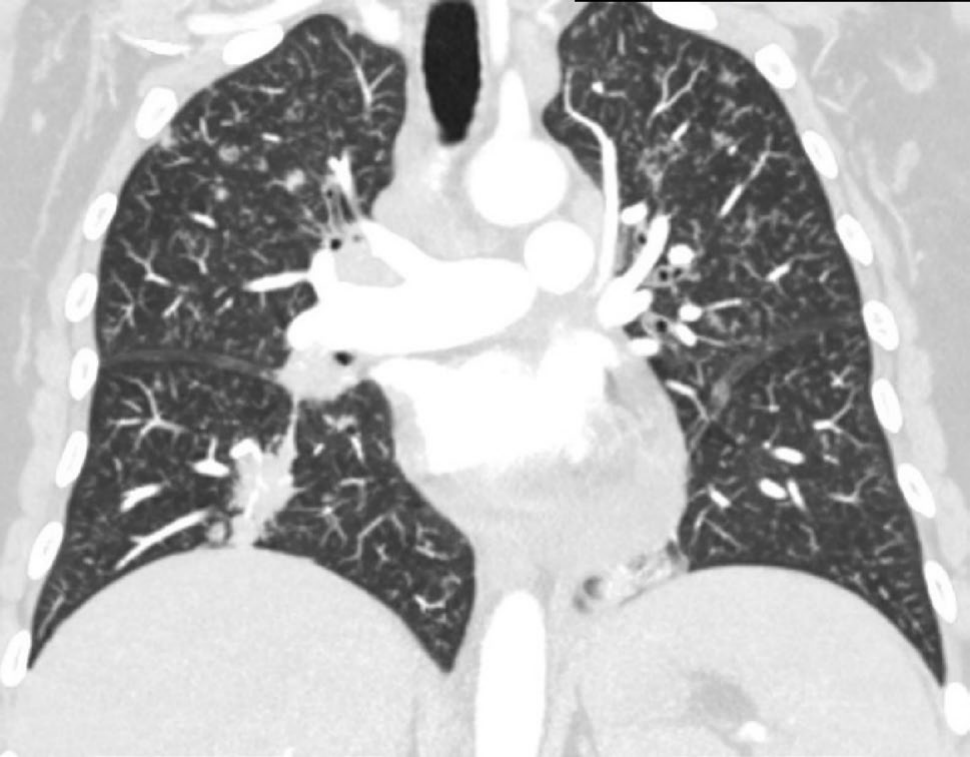
Coronal reconstruction of contrast-enhanced chest CT demonstrates multiple pseudonodular lesions in both lung fields, predominantly in the upper lobes, with consolidation in the right lower lobe

**Fig. 4:**
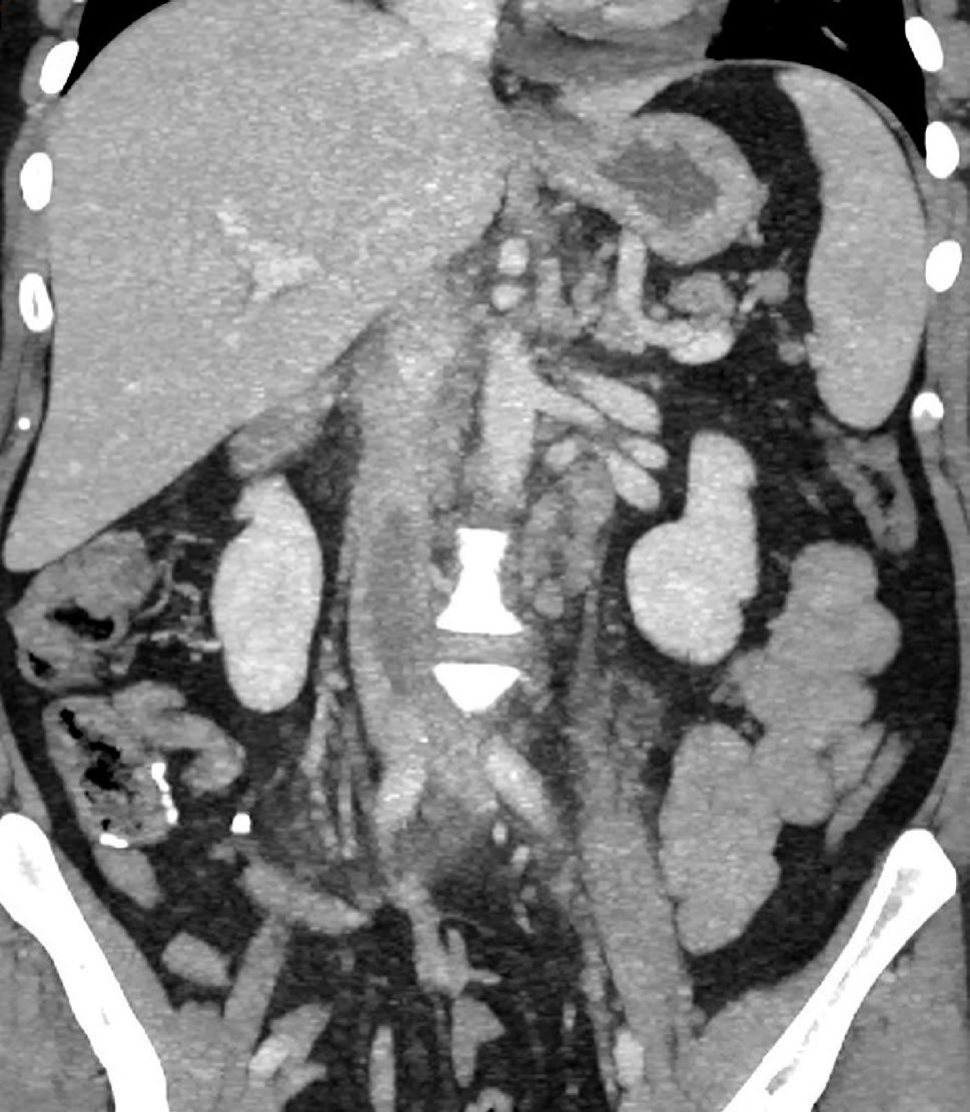
Coronal contrast-enhanced abdominopelvic CT image demonstrates an extensive thrombus within the inferior vena cava, extending into the left common iliac vein, accompanied by retroperitoneal lymphadenopathy and associated inflammatory changes

Due to persistent fever and new-onset respiratory symptoms, including dyspnea and cough, imaging was extended. A chest CT and PET-CT demonstrated bilateral ground-glass opacities, hepatosplenomegaly, and extensive hypermetabolic lymphadenopathy (Figs. 2, 3 and 4). A repeated IGRA showed a clear conversion to positive (5.09 IU/mL and 4.87 IU/mL). Sputum and bronchoalveolar lavage samples were positive for *Mycobacterium tuberculosis* (Xpert® MTB/XDR PCR and culture). A supraclavicular lymph node biopsy confirmed tuberculous involvement and ruled out lymphoproliferative disorders. A retrospective review of the 2021 surgical specimens and subsequent biopsies re-confirmed the initial IBD diagnosis without evidence of prior tuberculosis.

The clinical event was established as disseminated tuberculosis, appearing as a new primary infection rather than a reactivation of prior disease. A four-drug antituberculous regimen was initiated. Although the patient required 12 days of invasive mechanical ventilation, she was successfully stabilized. Anticoagulation management was complicated by a significant drug interaction between rifampicin and acenocoumarol, necessitating careful monitoring.

**Fig. 5:**
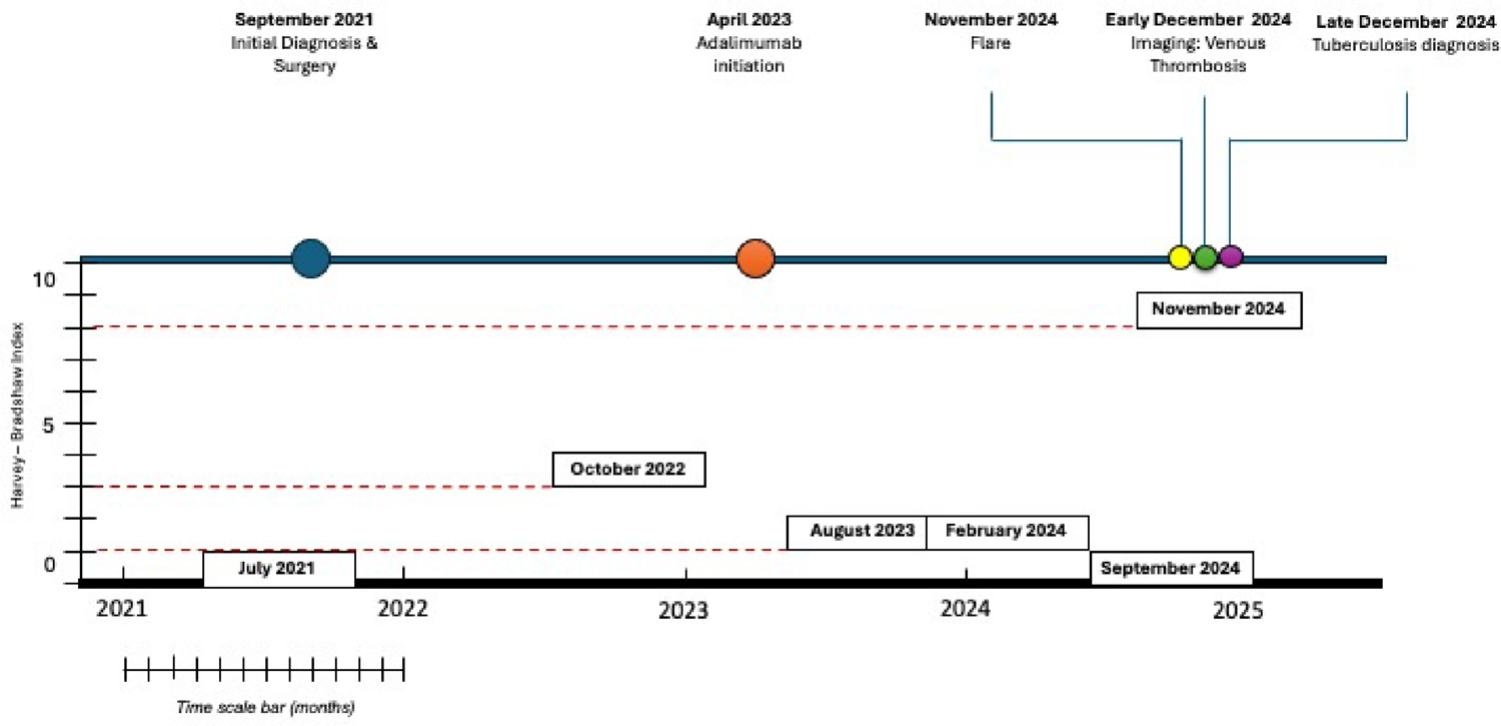
Chronologically proportional clinical timeline of the patient’s disease course. The timeline illustrates key clinical milestones from initial diagnosis to the final infectious complication. A longitudinal assessment of disease activity according to the Harvey–Bradshaw Index (HBI) is incorporated into the figure. The patient was in clinical remission in July 2021 (HBI 0), exhibited mild disease activity in October 2022 (HBI 3), and remained in clinical remission or minimal activity in August 2023 (HBI 1), February 2024 (HBI 1), and September 2024 (HBI 0). At the time of the disease flare in November 2024, the HBI increased to 8, consistent with a significant clinical exacerbation. Not to scale vertically; scale applies only to time

The patient was discharged after 44 days and is currently completing the consolidation phase of antituberculous therapy. Adalimumab was discontinued; however, the patient remains in clinical and radiological remission under close monitoring without active IBD treatment (Fig. 5). Written informed consent for publication was obtained from the patient.

## Discussion

This case highlights the diagnostic challenges in immunosuppressed patients with Crohn’s disease (CD). The initial constellation of fever, gastrointestinal symptoms, and extensive venous thrombosis (VTE) closely mimicked an IBD flare. These findings prompted a reassessment of the working diagnosis. Retrospectively, while the jejunal involvement could be consistent with active CD, its presentation alongside disseminated disease suggests it may also have been a manifestation of intestinal tuberculosis (TB). Given the significant radiological overlap between these entities, a definitive attribution remains complex. Furthermore, the clinical course**—**characterized by a long treatment-free interval and a documented IGRA conversion—supports the diagnosis of a new primary infection rather than a reactivation of latent disease.

The incidence of active TB is closely linked to anti-TNF therapy and regional prevalence [12, 13]. While anti-TNF agents significantly increase the risk of TB in IBD patients [14], recent data show low screening conversion rates in some cohorts [15, 16]. In Spain, prevalence among anti-TNF users ranges from 4.5 to 12.5% [17, 18], fueling debate regarding the necessity of routine re-testing in patients with initial negative results [19, 20]. Although TB risk may vary across different agents [21], no significant difference has been consistently observed between anti-TNF monotherapy and combination regimens [22]. Notably, de novo tuberculosis during anti-TNF therapy has been documented with both infliximab and adalimumab [18, 21, 23–33]. Meta-analyses have shown that anti-TNF therapy doubles the risk of tuberculosis infection in IBD [6, 7]. Tuberculosis can still develop during TNF antagonist therapy despite latent TB treatment [34, 35]. De novo tuberculosis during anti-TNF therapy has been predominantly associated with infliximab, followed by adalimumab.

Anti-TNFα therapy can mask typical tuberculosis presentations, leading to a higher incidence of extrapulmonary disease, as observed in our case. While typical TB often presents with insidious extrapulmonary disease [30, 36], including increased extrapulmonary involvement and a more insidious onset [31]. In our patient, the initial absence of respiratory symptoms and the presence of VTE obscured the diagnosis, underscoring that classic pulmonary complaints may be delayed or absent in this population [30, 36]. Consequently, a definitive diagnosis required invasive procedures, such as bronchoscopy with bronchoalveolar lavage and supraclavicular lymph node biopsy. This is consistent with reports indicating that patients developing TB on biologics often present with higher rates of non-respiratory symptoms and lower acid-fast bacilli smear positivity compared to the general population.

The extensive VTE initially contributed to diagnostic confusion. While IBD is a recognized prothrombotic state [5], TB also induces a hypercoagulable state through systemic inflammation and endothelial dysfunction [37, 38]. Current evidence suggests that anti-TNF agents may have a protective effect against VTE in CD, whereas chronic systemic steroid use increases this risk [39] [40]. Notably, chronic steroids were not part of this patient’s maintenance regimen prior to admission; although corticosteroids were administered acutely upon presentation for a suspected flare, the patient lacked long-term steroid-induced immunosuppression.

Managing this case was further complicated by the interaction between rifampicin—a potent cytochrome P450 inducer—and acenocoumarol, necessitating frequent dose adjustments. This highlights the critical need to consider complex drug-drug interactions when treating disseminated TB in patients with comorbidities.

In conclusion, this case serves as a clinical warning regarding the atypical presentation of disseminated TB in CD patients on anti-TNF therapy. The mimicry of IBD complications can significantly delay diagnosis. To our knowledge, this is the first reported case documenting the concurrent triad of Crohn’s disease, disseminated tuberculosis, and extensive venous thrombosis.
